# PollAgri: a new dataset on insect floral visitors of crops in France (first version: from 2004 to 2024).

**DOI:** 10.1016/j.dib.2026.113161

**Published:** 2026-08-13

**Authors:** Lucas Aubouin, Angélique Lécrivain, Benjamin Coussy, Stan Chabert, Laurent Guilbaud, Mickaël Henry, Nicolas Morison, Bernard Vaissière, Orianne Rollin, Willem Proesmans, Elsa Blareau, Isabelle Dajoz, Fabrice Requier, Marie-Sylvie Auffret, Elise Breton, Candy Cadet, Isabella Dellaica, Christel Dupuy, Auriane Eisenberg, Adrien Fauroux, Cécile Joly, Arlette Keller, Elena Lemercier, Franck Leprince, Anne Matte, Lise Maraval, Christophe Menoux, Bérénice Obert, Christophe Philippe, Julien Plaisse, Marie-Hélène Poussin, Christophe Reboud, Sabine Saly, Nathalie Serres, Armand Surrel, Eric Van de Pitte, Cassandre Murail-Zimmermann, Audrey Alignier, Lucie Schurr, Stéphanie Aviron, Colin van Reeth, Bertrand Schatz, Benoît Geslin

**Affiliations:** a CEFE, CNRS (UMR 5175), Université de Montpellier, EPHE, IRD, 1919 Route de Mende, 34293 Montpellier, France; b FNAMS, Impasse du Verger, Brain sur l'Authion, 49800, Loire-Authion; c Univ Toulouse, INRAE, CNRS, LIPME, 24 chemin de Borde Rouge, Auzeville CS 52627, 31326 Castanet-Tolosan, France; d INRAE, UR 406 Abeilles et Environnement, 228 Route de l’Aérodrôme, Site Agroparc, CS 40509, 84914 AVIGNON Cedex 9, France; e ITSAP, Institut de l’abeille, Domaine Saint-Paul, 228 Route de l’Aérodrôme, Site Agroparc, CS 40509, 84914 AVIGNON Cedex 9, France; f Institut Agro Dijon, INRAE, UMR Agroécologie, Université de Bourgogne Europe, 28 Boulevard Dr. Petitjean, BP 87999, 21079 Dijon Cedex, France; g Sorbonne Université, Université Paris Cité, Univ Paris Est Créteil, CNRS, IRD, INRAE, Institut d'écologie et des sciences de l'environnement de Paris, IEES, 4 place Jussieu 75005 Paris, France; h Université Paris-Saclay, CNRS, IRD, UMR Évolution, Génomes, Comportement et Écologie, 12 route 128, 91198 Gif-sur-Yvette, France; i Bergerie nationale de Rambouillet, Parc du château, CS40609, 78514, Rambouillet Cedex; j CITERES - UMR 7324 - Cités, Territoires, Environnement et Sociétés, 33 Allée Ferdinand de Lesseps BP 60449, 37204 Tours cedex 3, France; k UMR 0980 BAGAP, L’Institut Agro Rennes-Angers-ESA-INRAE, 65 rue de Saint Brieuc, Rennes Cedex 35042, France; l Université Paris Cité (UPC), Sorbonne Université (SU), Université Paris Est Créteil (UPEC), CNRS, IRD, INRAE, Institut d'écologie et des sciences de l'environnement de Paris, IEES Paris, 4 place Jussieu 75005 Paris, France; m CREA Mont-Blanc, 67 Lacets du Belvédère, 74400 Chamonix-Mont-Blanc, France; n Université de Rennes, UMR 6553 Ecosystems, Biodiversity, Evolution, CNRS, 263 avenue du Général Leclerc, 35042 Rennes, France

**Keywords:** Bees, Wild bees, Syrphids, Butterflies, Beetles, Pollinators, Agriculture, Communities

## Abstract

Animal-mediated pollination is essential to our food system: around 75% of crops worldwide depend, at least partially, on floral visitations by animals for their production. Crop pollinators, primarily insects, display an increasing role in global crop yields as the overall surface of animal pollinated crops grows. Yet, insects experience a massive decline worldwide, and pollinators such as bees and dipterans make no exception, potentially threatening crop yield and food security. Identifying insect floral visitors is necessary to build a solid overview of crop-associated pollinators, to enlighten their ecological requirements and the drivers of their subsistence in agricultural landscapes. Surprisingly, while pollinators are central to our food production, basic information on the identity of insect floral visitors remains very limited for many cultivated plants. Here we introduce the first version of a centralised and standardised database called PollAgri, that gathers interaction data between insects and flowers of entomophilous crops in a major agricultural country, Metropolitan France. The database aggregates data thanks to a collaboration with the CropPol project, that provides information on crop pollination worldwide, and a call for contributions to our network of researchers and naturalists. Aggregated data was collected from May 2004 to September 2024 from multiple regions in France. PollAgri gathers 80,935 direct interactions involving 417 insect taxa at various taxonomic ranks, from order to species, for 51 species of cultivated plants. This database is a first step towards the identification of floral visitor communities specific to each crop species, a fundamental requirement to better assess the contribution of insects to crop yield. The database helps pinpoint crops, regions and seasons that are under-sampled, orienting future fieldwork. Furthermore, completed with ecological data on these species, PollAgri will help target pollinator-friendly agricultural practices. Hopefully, the database will stimulate research on crop flower visitors, and encourage the community of naturalists and researchers to share their data and help mitigate pollinator decline and its impacts on both ecosystem stability and food security.

Specifications Table

**Table:** 

Subject	Earth & Environmental Sciences
Specific subject area	Floral visitors of crops
Type of data	Table in CSV format Metadata in EML format
Data collection	A call for observations of insect floral visitors of crops in France was launched to 72 naturalists and researchers from our network, and a collaboration with the CropPol project, an open database on crop pollination was initiated to select variables of interest. All shared data was carefully scrutinized, and only direct interactions of an insect with the flower of a cultivated plant were included in PollAgri. This enabled the compiling of data produced from 17 projects (research, citizen science and technical institutes). Retrieved data was centralised and standardised using ecological standards (TaxRef for species names, French statistical office for agricultural data for crop categories).
Data source location	Institution: Primary data: CEFE (UMR 5175 CNRS), 1919 route de Mende, Montpellier, Hérault, Occitanie. Country : France - 46.6° N, 1.9° E Secondary data were obtained from the CropPol database (Allen-Perkins, A. et al., 2022. CropPol: A dynamic, open and global database on crop pollination. Ecology 103, e3614. https://doi.org/10.1002/ecy.3614).
Data accessibility	Repository name: InDoRES Data identification number: https://doi.org/10.48579/PRO/YV7MXG Direct URL to data: https://data.indores.fr/dataset.xhtml?persistentId=doi:10.48579/PRO/YV7MXG
Related research article	*None*

## 1 Value of the Data

• Identifying insect floral visitors of crops and their assemblages crop-by-crop is fundamental to understand the links between cultivated plants and their pollinators.
• To our knowledge, this is the first comprehensive database of crop floral visitors within France, the first agricultural exporter of the European Union. The database also takes part in a global research effort on crop pollination at the international scale (CroPol).
• First of all, the data is valuable to fulfil the basic gaps of knowledge on assemblages of floral visitors of crops, crop-by-crop. Along with ecological data on these insects, PollAgri will greatly contribute to building a pollinator-friendly frame of the ecological transition of agriculture, by allowing an accurate definition of ecological requirements specific to crop pollinators. As food production mostly relies on these insects, this will also be central to ensure sustainable crop productivity. Furthermore, it will help assess changes in community structures of floral visitors of crops along spatial and temporal gradients and will be central to characterise the dependence of crops to entomological diversity. By extension, PollAgri will contribute to better evaluate crops’ sensitivity to environmental pressures.
• The data also permits the elaboration of a state of the art on data availability on crop insect visitors. It will namely help identify crops with few or no available data, crops with insufficient or inadequate geographic and temporal coverage, or gaps in taxonomical resolution of insects. These elements need to be considered key priorities to refine global fieldwork in the future, in order to support pollinator conservation in agroecosystems.
• The crop plants examined in this work are grown in many other European countries and the data presented here may thus be applicable to bordering countries. PollAgri will hopefully encourage comparisons of pollinators and visiting insects across different contexts. The dataset promotes collaboration between researchers, technical institutes and citizens, and contributes to the dissemination of a large amount of open research data on this critical topic.

## 2 Background

Modern agriculture faces a major challenge: the area of pollinator-dependent crops and the demand for insect pollination are increasing globally [1,2], whereas an ever-growing body of scientific literature highlights the alarming decline of insect pollinators worldwide [[3], [4], [5]]. As a result, many crops endure pollination deficits, weakening yield, fruit and seed quality, and threatening food security [[6], [7], [8], [9]]. Some rare studies have explored the identity of these insect floral visitors on the international scale [10] or regionally [[11], [12], [13]]. They showed that different crops are associated to different insect assemblages, indicating that food production relies on crop-specific insect communities. Understanding their composition and ecological requirements is pivotal to build a pollinator-friendly transition of agriculture, and to sustain food security. Surprisingly, most of agricultural countries such as France still lack reference lists of floral visitors of their crops. Here, we compiled available data on insect floral visitors of crops within France. We mobilised a large network of scientists to centralise flower-insect interaction data, defined as insects contacting the reproductive structures of a flower (pistil or stamens). Data provided here are fully interoperable and reusable within international databases. PollAgri will hopefully stimulate research leading to a higher conservation of insects in agricultural landscapes.

## 3 Data Description

Core data are stored in a table in the *pollAgri.csv* file. The file is deposited in the InDoRES data repository at https://doi.org/10.48579/PRO/YV7MXG [14]. This table is structured following four groups of variables: 1) information on the taxonomy of the floral visitor, beginning with POLL_, 2) information on the identity and characteristics of the crop, beginning with CROP_, 3) information on the study that generated the data (location, dates, methodology), beginning with STUDY_, and 4) the reference associated to the publication of the data (Table 1).

**Table 1:** Description of the 49 variables included in the pollAgri.csv file.

Column	Variable	Description	Group of variables
1	ID	ID of the data	Identification number of the data
2	POLL_LB_NOM	Floral visitor name as given is the original study	Information on the floral visitor
3	POLL_VALID_NAME	Floral visitor valid name following the TAXREF V18 taxonomy	
4	POLL_CD_NOM	Floral visitor TaxRef ID	
5	POLL_RANK	Floral visitor taxonomic rank	
6	POLL_REALM	Floral visitor realm	
7	POLL_PHYLUM	Floral visitor phylum	
8	POLL_CLASS	Floral visitor class	
9	POLL_ORDER	Floral visitor order	
10	POLL_FAMILY	Floral visitor family	
11	POLL_GENUS	Floral visitor genus	
12	POLL_SUBFAMILY	Floral visitor subfamily	
13	POLL_TRIBE	Floral visitor tribe	
14	POLL_SEX	Floral visitor sex	
15	POLL_CASTE	Floral visitor caste	
16	CROP_LB_NOM	Name of the crop as given in the original study	Crop information
17	CROP_VALID_NAME	Crop valid name following the TAXREF V18 taxonomy	
18	CROP_CD_NOM	Crop TaxRef ID	
19	CROP_RANK	Crop taxonomic rank	
20	CROP_REALM	Crop realm	
21	CROP_PHYLUM	Crop phylum	
22	CROP_CLASS	Crop class	
23	CROP_ORDER	Crop order	
24	CROP_FAMILY	Crop family	
25	CROP_GENUS	Crop genus	
26	CROP_SUBFAMILY	Crop subfamily	
27	CROP_TRIBE	Crop tribe	
28	CROP_COMMON_NAME	Crop common name (in French)	
29	CROP_AGRESTE_ CATEGORY	Crop primary category following the AGRESTE classification	
30	CROP_MUNICIPALITY	Municipality where the data were produced	
31	CROP_POSTAL_CODE	Postal code of the municipality where the data were produced	
32	CROP_DEPARTMENT	French department where the data were produced	
33	CROP_LATITUDE	Latitude associated to the data	
34	CROP_LONGITUDE	Longitude associated to the data	
35	CROP_CONTEXT	The physical or management context in which the crop was grown (field, park, garden, experimentation station)	
36	CROP_URBANISATION	Urbanisation type where the crop was located (rural; suburban; urban)	
37	CROP_CROP_TYPE	Crop type (arable farming; market gardening; meadow; orchard)	
38	CROP_SEED_PRODUCER	Was the crop cultivated to produce seeds?	
39	CROP_MANAGEMENT	Crop management (organic; conventional; integrated pest-management)	
40	CROP_FIELD_AREA	Area of the field, in m^2^	
41	STUDY_DAY	Number of the day of the production of the data	Information on the original study
42	STUDY_MONTH	Month of the production of the data	
43	STUDY_YEAR	Year of the production of the data	
44	STUDY_TIME	Time the data was generated	
45	STUDY_METHOD	Insect sampling methodology (net capture; camera; on sight; falcon tube)	
46	STUDY_PROTOCOL	Insect sampling methodology (transect; quadrat)	
47	STUDY_SAMPLED_AREA	Sampled area, in m^2^	
48	STUDY_SAMPLING_DURATION	Sampling duration	
49	SOURCE	Bibliographic reference of the data	Reference

One data is stored within one row. As a consequence, abundance information results in the multiplication of rows if one floral visitor has been observed multiple times on the same cultivated plant.

Metadata are stored within the *pollAgriMetada.xml* file using the Ecological Metadata Language [15]. The file describes the overall dataset and provides detailed information on every single variable and term used in the *pollAgri.csv* file. This standardised format will facilitate interoperability and integration of data contained in PollAgri into other international databases on biotic interactions such as Global Biotic Interactions [16] or within datasets on crop pollination [10].

The table contains 80,935 unique and direct interactions between an insect and the flower of a crop within the French Metropolitan area. These observations were retrieved from 17 references, representing large-scale studies shared by our network of researchers and naturalists (Table 2).

**Table 2:** List of selected sources, with studied crops and data acquisition methods.

Source	Studied crop species	Data acquisition method
Alignier et al., 2023	Faba bean, pea	On sight
Allen-Perkins et al., 2022	Apple, sunflower, rapeseed, leak	Net captures, on sight
Aviron, S. et al., 2023	Faba bean, pea	On sight
Blareau 2025.	Strawberry, raspberry	On sight
Carré et al., 2009	Melon	Net captures
Chabert et al., 2022	Sunflower	On sight
Comm. Pers. Marie-Sylvie Auffret (data from the citizen science program PHYT’ABEILLES)	Alfalfa	Net
Coussy 2015	Carrot	Net capture, on sight
Coussy 2019	Carrot	Camera, on sight
Fortel et al., 2016	Strawberry	Net capture
Murail-Zimmermann et al., 2026	Rapeseed	Falcon tube
Pers. Comm. from Laurent Guilbaud, Bernard Vaissière & Mickaël Henry (observations from the INRAE laboratory)	Rapeseed, buckwheat, cabbage, chicory, sunflower, apple, pea, pear, sweet cherry, raspberry, faba bean, fodder crops, aromatic plants	Net capture
Proesmans et al., 2024	Aromatic plants, rapeseed, black mustard, quince, fennel, soybean, sunflower, apple, pea, pear, faba bean	Net capture
Rollin et al., 2013	Rapeseed, carrot, sunflower, flax, aromatic plants, fodder plants	Net capture
Schurr 2022	Fennel,	Net capture
Surrel et al., 2026	Rapeseed, pumpkin, sunflower, pea, faba bean	Net capture
Van Reeth 2026	Blackcurrent, raspberry, fodder crops	Net capture

**Fig. 1:**
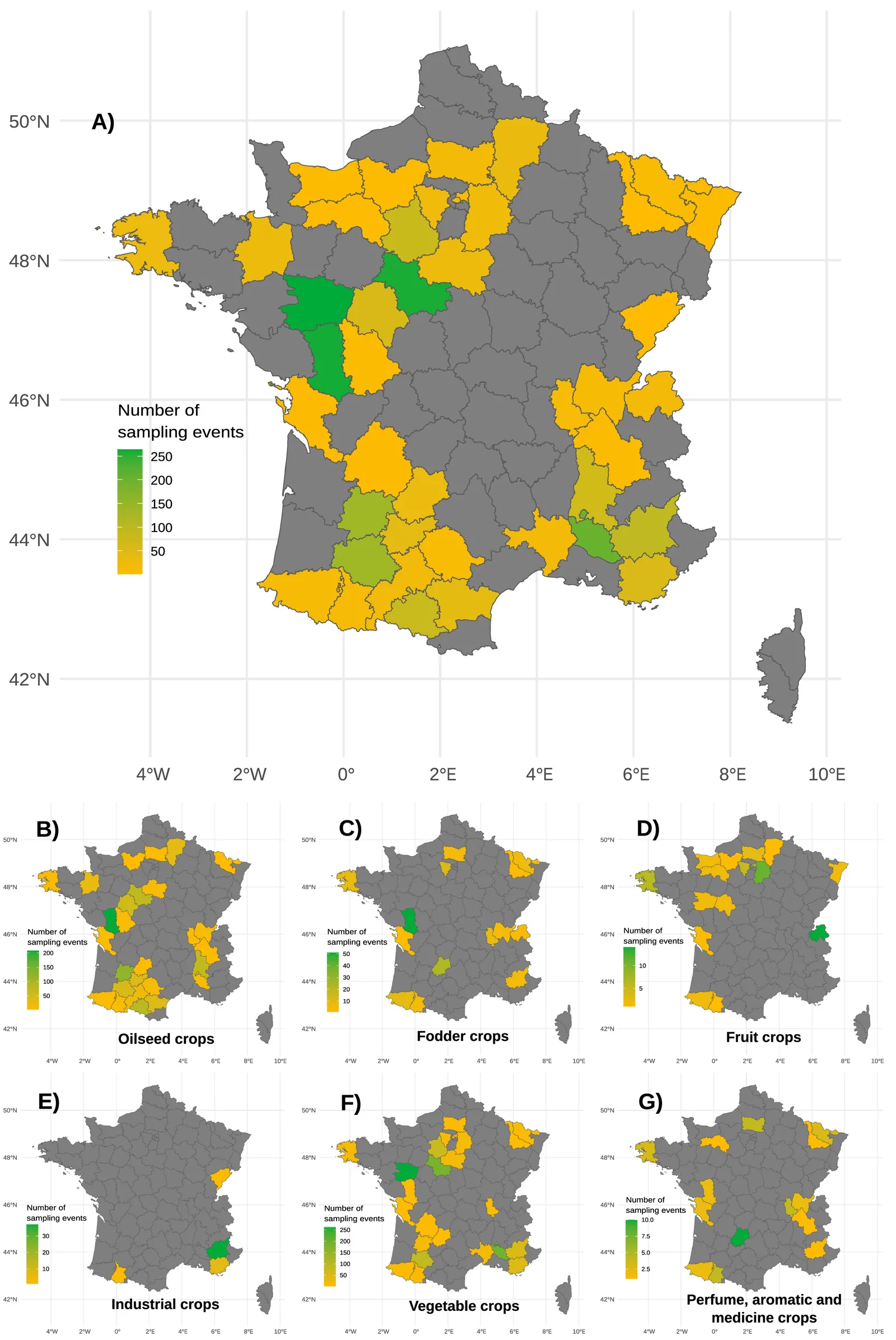
Geographic distribution of sampling events within the French metropolitan territory for the whole dataset and for each crop category. In grey, departments with no sampling event.

Documented data were collected throughout 2,189 sampling events. The data shows an uneven sampling effort across the country, with six departments gathering more than 100 sampling events: Maine-et-Loire (264 sampling events), Deux-Sèvres (261), Loir-et-Cher (259), Vaucluse (204), Gers (137) and Lot-et-Garonne (137). Out of the 96 departments in Metropolitan France, only 44 were sampled (Fig. 1).

**Fig. 2:**
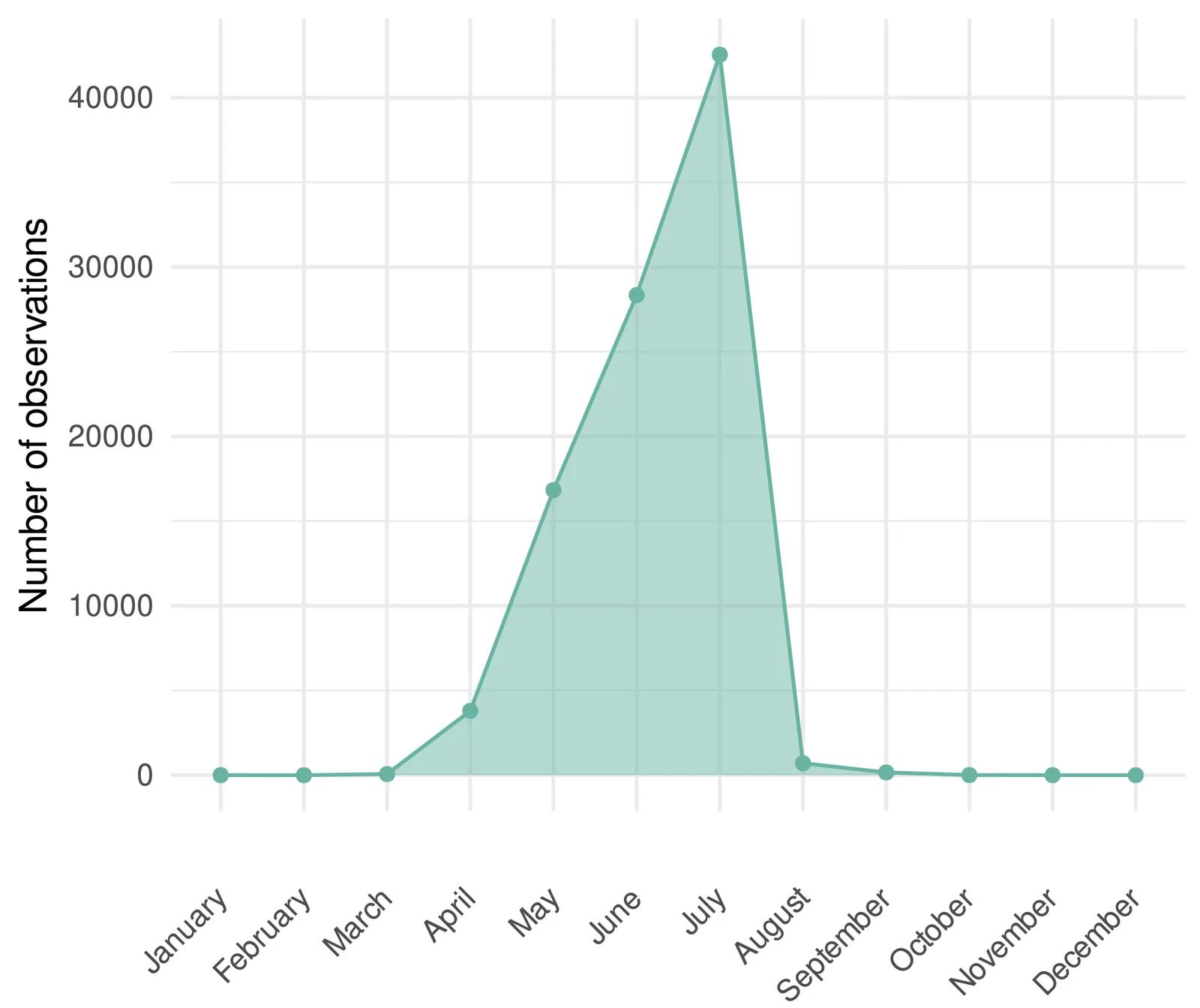
Monthly temporal coverage of the data included in PollAgri. All years are considered, from 2004 to 2024.

Data were produced from May 2004 to September 2024. The majority of the data were collected between May and July, with 16,828 interactions between an insect and the flower of a crop recorded in May, representing 20.79% of all data, 23,352 interactions in June (28.85%) and 36,021 in July (44.51%) (Fig. 2). No data was collected in January, February, November nor December.

The database identifies 417 floral visitor taxa at various taxonomic levels, from species to order, mostly composed of hymenopterans (Table 3). The most observed floral visitor species was *Apis mellifera*, with 47,888 observations, representing 59.1% of the total number of observations. We also gathered 12,307 observations of *Bombus terrestris* (15.2%), 1,978 observations of *Bombus lapidarius* (2.44%) and 1,177 observations of *Eristalis tenax* (1.45%), a non-Apoidea species.

**Table 3:** Number of observations per order of floral visitor.

Order	Number of observations	Proportion
Hymenoptera	74,034	91.47%
Diptera	6,706	8.29%
Coleoptera	107	0.13%
Hemiptera	52	< 0.1%
Lepidoptera	22	< 0.1%
Neuroptera	8	< 0.1%
Araneae	3	< 0.1%
Thysanoptera	2	< 0.1%
Orthoptera	1	< 0.1%
**TOTAL**	**80,935**	**100%**

Observations were made on 51 cultivated plants. The most sampled crop was *Helianthus annuus* with 29,509 observations, followed by *Daucus carota* (28,476 observations), *Allium porrum* (15,529), *Malus domestica* (2,284), *Brassica napus* (1,971) and *Medicago sativa* (1,288) (Table 4).

**Table 4:** Number of observations per crop (only the 10 most sampled crops are displayed).

Crop common name	Crop scientific name	Number of observations
Sunflower	*Helianthus annuus L., 1753*	28,509
Carrot	*Daucus carota L., 1753*	28,476
Leek	*Allium porrum L., 1753*	15,529
Apple	*Malus domestica (Suckow) Borkh., 1803*	2,284
Oilseed rape	*Brassica napus L., 1753*	1,971
Alfalfa	*Medicago sativa L., 1753*	1,288
Raspberry	*Rubus idaeus L., 1753*	671
Cantaloupe melon	*Cucumis melo L., 1753*	528
Black mustard	*Brassica nigra (L.) W.D.J.Koch, 1833*	244
Fava bean	*Vicia faba L., 1753*	226

## 4 Experimental Design, Materials and Methods

### 4.1 Data definition

The aim of PollAgri is to inform on insect assemblages associated to crop plants within the French Metropolitan area. As it can be challenging to investigate if insects actually pollinate a crop [17], we use the term *floral visitors* to name the insects that forage on crop flowers. Insect taxonomy is aligned with the national taxonomic register TAXREF [18].

One *data* is characterised by a quadruplet of information:

1) information on the taxonomy of the floral visitor, from order to species when available,
2) information on the taxonomy of the cultivated plant (at least identified at the species level, and when possible, at the variety level), on the crop type or on agricultural management,
3) details on the original study such as geographical information, temporal information, and sampling methods,
4) the associated reference. We did not record yield information as these data are out of the scope of this work.

### 4.2 Crop definition

We define *crop* as every plant that is cultivated in order to be at least partly harvested for consumption (human or livestock food, seed production, essential oil production) or industrial purposes (e.g. textile, fuel, stimulants). We excluded here ornamental species. We focused our research on entomophilous crops, i.e. that are mainly pollinated by insects. As a result, cereals were excluded from our database. The plant taxonomy is consistent with the national taxonomic register TAXREF of France [18]. Each cultivated plant was categorised according to the French statistical office for agricultural data [19].

### 4.3 Interaction definition

An *interaction* is defined as a contact between an insect taxon (from various taxonomic levels, from species to order) and the flower of a cultivated plant. Consequently, data from net captures and visual or photographic observations of floral visitors are integrated into the database, as they guarantee direct interactions between insects and flowers. In contrast, data from pan traps are not used as they are not proof of such an interaction.

### 4.4 Sampling event definition

Sampling effort can be translated into *sampling events*. One sampling event is defined by a triplet of information: the date, the site and the project for which the data were produced.

### 4.5 General workflow

To gather the data, we first initiated a collaboration with the CropPol project, an open and global database on crop pollination. With the authors' agreement, we extracted a subset of observations specifically collected within French territory. We then issued a call for primary data to 72 naturalists and researchers from our network (i.e. the POLLINECO scientific network and complementary collaboration networks). We retrieved and centralised all the shared data using a data template that we specifically adapted to our needs. After this, we formatted all datasets using ecological standards such as the French taxonomic register TaxRef and crop categorisations established by the French statistical office for agricultural data. Finally, we excluded data that did not meet the awaited requirements (indirect interaction or uncultivated context).

All data in this first version of PollAgri were generated by the authors of this paper or their research institutes, except for published data extracted from the CropPol database.

### 4.6 Sampling protocols

Data was produced from multiple projects on insect communities in crops using various methodologies (Table 2). We identify four distinct protocols. First, specimens were identified on sight, by waiting on single flowers or following transects. Secondly, specimens were captured with nets following transects or within quadrats. Third, specimens were photographed by waiting on single flowers, or within quadrats. Finally, specimens were directly captured with falcon tubes on the flowers. In all cases, when specimens were captured, they were then identified under a binocular microscope.

### 4.7 Further improvements

The work we provide here is the first version of an evolving database that will be regularly implemented with new data on crop floral visitors produced by the community of naturalists and researchers on the French Metropolitan territory. In the future, we especially intend to conduct a systematic literature survey and collaborate with citizen science projects such as SPIPOLL (Photographic survey of pollinators) in order to gather as much data as possible.

## Limitations

Although PollAgri gathers a large amount of observations on crop floral visitors, many entomophilous crops still require investigation to better represent cultivated crops in France. Moreover, already documented crops need to be further studied to build representative insect assemblages.

Fig. 1 reveals an overall uneven geographical coverage. As crop-associated insect assemblages may differ among regions, departments that were not sampled need to be investigated to improve representativeness. Furthermore, future sampling coverage should be consistent with the distribution of crops over the national area.

A large number of crop-visiting insects were identified to a taxonomic level higher than the species, which is the gold standard for faunistic surveys [20]. It is of utmost importance to more accurately identify observed individuals in future fieldwork, and to tend towards species-level identifications.

Fig. 2 shows that fieldwork mostly took place during summertime. As many other crops bloom in early Spring (e.g. almond, apricot), we lack information on floral-visitor assemblages for these crops. We also lack information on nocturnal flower visitors .

Finally, as we gathered observations from diverse protocols, data are only partially standardised. Future fieldwork will need to follow harmonised methodologies.

## Ethics Statement

The authors have read and follow the ethical requirements for publication in Data in Brief and confirm that the current work does not involve human subjects, animal experiments, or any data collected from social media platforms.

## Credit Author Statement

**Lucas Aubouin**: Conceptualization, Methodology, Validation, Investigation, Data curation, Writing - Original draft, Visualization; **Angélique Lécrivain**: Investigation, Data curation, Writing - Original draft, Visualization; **Marie-Sylvie Auffret**: Resources; **Elsa Blareau**: Resources; **Stan Chabert**: Resources; **Benjamin Coussy**: Resources; **Isabelle Dajoz**: Resources; **Laurent Guilbaud**: Resources; **Mickaël Henry**: Resources; **Cassandre Murail-Zimmermann**: Resources; **Willem Proesmans**: Resources; **Fabrice Requier**: Resources; **Lucie Schurr**: Resources; **Elise Breton**: Resources; **Candy Cadet**: Resources; **Isabella Dellaica**: Resources; **Christel Dupuy**: Resources; **Auriane Eisenberg**: Resources; **Adrien Fauroux**: Resources; **Cécile Joly**: Resources; **Arlette Keller**: Resources; **Elena Lemercier**: Resources; **Franck Leprince**: Resources; **Anne Matte**: Resources; **Lise Marval**: Resources; **Christophe Menoux**: Resources; **Bérénice Obert**: Resources; **Christophe Philippe**: Resources; **Julien Plaisse**: Resources; **Marie-Hélène Poussin**: Resources; **Christophe Reboud**: Resources; **Sabine Saly**: Resources; **Nathalie Serres**: Resources; **Armand Surrel**: Resources; **Eric Van de Pitte**: Resources; **Bertrand Schatz**: Conceptualization, Methodology, Supervision, Project administration, Funding acquisition; **Benoît Geslin**: Conceptualization, Methodology, Supervision, Project administration, Funding acquisition.

## Data Availability

DataversePollAgri: a dataset on insect floral visitors of crops (Original data)
